# Serum concentrations of Cu, Se, Fe and Zn in patients diagnosed with pancreatic cancer

**DOI:** 10.1186/1897-4287-13-S1-A14

**Published:** 2015-09-09

**Authors:** Marcin Lener, Anna Wiechowska-Kozlowska, Rodney J Scott, Magdalena Muszynska, Jozef Kladny, Piotr Waloszczyk, Anna Rutkowska, Grzegorz Sukiennicki, Tomasz Gromowski, Katarzyna Jaworska-Bieniek, Thierry van de Wetering, Katarzyna Kaczmarek, Anna Jakubowska, Jan Lubinski

**Affiliations:** 1 Department of Genetics and Pathology, Pomeranian Medical University in Szczecin, International Hereditary Cancer Center, Szczecin, Poland; 2 Division of Health Care Ministry of Internal Affairs and Administration in Szczecin, Poland; 3 Discipline of Medical Genetics, School of Biomedical Sciences, Faculty of Health, University of Newcastle and The Hunter Medical Research Institute, Newcastle, New South Wales 2308, Australia; 4 Read – Gene, S.A., Grzepnica, Poland; 5 Department of General and Oncological Surgery, Pomeranian Medical University, Szczecin, Poland; 6 Regional Hospital for Lung Diseases, Szczecin-Zdunowo, Poland

## Introduction

Pancreatic cancer is the eighth most commonly diagnosed cancer in the developed world and has one of the worst prognoses of any malignancy with 98% succumbing to their disease within 5 years. Little is known about the etiology of the disease despite significant new insights into the mutation signatures common to this disease. The antioxidants, including vitamins C, E, Se, Zn might prevent pancreatic cancer. In the current study we have examined the levels of Cu, Fe, Zn and Se in a moderately sized pancreatic cancer population and compared it to a healthy age-matched population.

## Material and methods

A total of 48 pancreatic cancer patients and 48 aged-matched healthy controls were enrolled in the study after providing informed consent. The patients with pancreatic cancer were enrolled to the study from the Hospital of the Ministry of Internal Affairs and Administration in Szczecin, Poland. For each pancreatic cancer patient included in this study an unaffected individual registered in International Hereditary Cancer Center, Pomeranian Medical University of Szczecin, was used as a control.

Each person enrolled in the study donated ~10 ml EDTA blood for sufficient serum to be isolated and examined for the elements Cu, Se, Fe and Zn.The level of Cu, Fe, Se, and Zn, in the serum was determined by mass spectrometry Inductively Coupled Plasma Mass Spectrometry (Elan DRC-e, PerkinElmer).

## Results

**Table 1:** Association between Se serum concentration and risk of pancreatic cancer

Cancer site	Quartile	Selenium concentration range (*µg/l)*	No. of cancer / controls
**Pancreatic cancer**	**I**	29.87-56.00	**22/2**

	**II**	56.43-68.47	11/13

	**III**	68.57-80.49	11/13

	**IV**	80.73-149.22	**4/20**

I vs II: p=0.013. OR = 13.000

I vs III: p=0.013. OR = 13.000

I vs IV, p< 0.0001, OR = 55.000

**Table 2:** Association between Cu serum concentration and risk of pancreatic cancer

Cancer site	Quartile	Copper concentration range (*µg/l)*	No. of cancer / controls
**Pancreatic cancer**	**I**	785.27-969.05	**3/21**

	**II**	977.76-1119.26	10/14

	**III**	1120.26-1363.22	12/12

	**IV**	1395.82-2420.82	**23/1**

II vs I: p = 0.049, OR = 5.000

III vs I: p = 0.0114, OR = 7.000

IV vs I: p< 0.0001, OR = 161.000

**Table 3:** Association between Fe serum concentration and risk of pancreatic cancer

Cancer site	Quartile	Iron concentration range (*µg/l)*	No. of cancer / controls
**Pancreatic cancer**	**I**	162.62-697.99	18/5

	**II**	714.61-947.19	10/13

	**III**	948.75-1193.73	7/16

	**IV**	1200.90-2987.68	11/12

I vs II: p = 0.0331, OR = 4.680

I vs III p = 0.0027, OR = 8.229

I vs IV: p = 0.0654, OR = 3.927

**Table 4:** Association between Zn serum concentration and risk of pancreatic cancer

Cancer site	Quartile	Zinc concentration range (*µg/l)*	No. of cancer / controls
**Pancreatic cancer**	**I**	306.12-739.81	15/9

	**II**	747.62-847.10	8/16

	**III**	848.81-995.08	13/11

	**IV**	1002.72-4896.90	12/12

I vs II: p = 0.082

I vs III: p = 0.770

I vs IV: p = 0.561

**Table 5:** Ratio Cu/Se

Cancer site	Quartile	Cu/Se concentration ratio	No. of cancer / controls
**Pancreatic cancer**	**I**	0.55-13.04	**3/21**

	**II**	13.08-16.35	6/18

	**III**	16.45-23.91	15/9

	**IV**	23.98-55.03	**24/0**

II vs I: p = 0.4614, OR = 2.333

III vs I: p = 0.0008, OR = 11.667

IV vs I: p < 0.0001, OR = 301

**Table 6:** Ratio Zn/Se

Cancer site	Quartile	Zn/Se concentration ratio	No. of cancer / controls
**Pancreatic cancer**	**I**	0.43-9.87	9/15

	**II**	9.97-13.20	6/18

	**III**	13.29-15.81	15/9

	**IV**	15.82-38.90	18/6

IV vs I: p = 0.018, OR = 5.000

III vs I: p = 0.148, II vs I: p = 0.534

**Table 7:** Ratio Fe/Se

Cancer site	Quartile	Fe/Se concentration ratio	No. of cancer / controls
**Pancreatic cancer**	**I**	0.63-10.56	12/11

	**II**	10.67-12.91	8/15

	**III**	12.93-18.28	11/12

	**IV**	18.29-41.84	15/8

I vs II: p = 0.372,

I vs III: p = 1.000,

I vs IV: p = 0.549

## Conclusions

1. There is a very strong correlation between the level of selenium, copper in serum and the risk of pancreatic cancers in the Polish population.

2. The Se, Cu level in serum and especially ratio Cu/Se may be a useful diagnostic tool of pancreatic cancer.

3. Further investigations are needed to determine if Cu/Se ratio can be used in:

a. differential diagnosis of pancreatic tumor (PT),

b. identification of causative factors of PT,

c. identification of prognostic factor of PC.

